# Whispers of decay: necrotic mediastinal lymphadenopathy due to melioidosis in a diabetic patient

**DOI:** 10.1093/omcr/omag032

**Published:** 2026-03-23

**Authors:** Rathan Kamath, Nitin Gupta, Anirudh Venkatesh, Muralidhar Varma, Praveen Kumar Tirlangi

**Affiliations:** Department of Infectious Diseases, Kasturba Medical College, Manipal Academy of Higher Education, Manipal, Udupi, Karnataka 576104, India; Department of Infectious Diseases, Kasturba Medical College, Manipal Academy of Higher Education, Manipal, Udupi, Karnataka 576104, India; Department of Infectious Diseases, Kasturba Medical College, Manipal Academy of Higher Education, Manipal, Udupi, Karnataka 576104, India; Department of Infectious Diseases, Kasturba Medical College, Manipal Academy of Higher Education, Manipal, Udupi, Karnataka 576104, India; Department of Infectious Diseases, Kasturba Medical College, Manipal Academy of Higher Education, Manipal, Udupi, Karnataka 576104, India

## Abstract

We report a case of melioidosis presenting as isolated necrotic mediastinal lymphadenopathy in a 60-year-old man from coastal Karnataka with poorly controlled diabetes (HbA1c 9.9%). Such a presentation, closely mimicking tuberculosis, is rarely described in the literature. Dual microbiological confirmation through sputum culture and endobronchial ultrasound (EBUS) guided lymph node aspiration established the diagnosis of *Burkholderia pseudomallei*. This case underscores the importance of considering melioidosis in the differential diagnosis of necrotic mediastinal lymphadenopathy, particularly in endemic regions and in diabetic patients. Early recognition and appropriate therapy remain crucial to prevent complications and improve outcomes in this potentially fatal but treatable infection.

## Case

**Figure 1:**
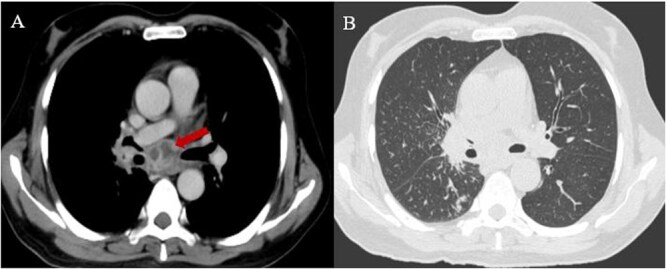
Multiple large necrotic mediastinal lymph nodes (A), along with centrilobular nodules in the superior segment of the right lower lobe (B).

A 60-year-old resident of coastal Karnataka, with poorly controlled diabetes (Glycosylated haemoglobin-9.9%), presented with a 25-day history of fever, chills, and a cough with minimal expectoration. He denied haemoptysis, chest pain, or shortness of breath. Initial investigations revealed mild anaemia (haemoglobin 11.9 g/dl), neutrophilic leucocytosis (total leukocyte count 19 400 cells/mm^3^ with 81% neutrophils), and elevated C-reactive protein (142.7 mg/dl). Liver and renal function tests were routine. Blood cultures were sterile, and no vegetation was detected on two-dimensional echocardiography. A contrast-enhanced chest computed tomography (CT) revealed multiple large necrotic mediastinal lymph nodes and centrilobular nodules in the superior segment of the right lower lobe (Fig. 1). With a suspicion of tuberculosis, sputum samples were sent for acid-fast staining, GeneXpert Ultra, and mycobacterial culture, all of which were negative. A sputum sample was sent for bacterial culture, which grew *Burkholderia pseudomallei*. Subsequent endobronchial ultrasound (EBUS)-guided aspiration of mediastinal lymph nodes was also positive for the same organism. The patient was started on intravenous meropenem (1 gram three times a day), with subsequent resolution of his fever within 8 days. Meropenem was continued for 14 days and then switched to oral co-trimoxazole (800/160, two tablets twice a day, for 4 months) for the continuation phase of treatment.

## Discussion

Melioidosis, a potentially fatal infection caused by *B. pseudomallei*, is primarily endemic in tropical regions of Southeast Asia, Northern Australia, and India [1, 2]. Clinical presentations vary widely, ranging from fulminant septic shock to a more indolent, chronic disease characterised by abscesses in multiple organs [3]. This broad spectrum often leads to diagnostic delays, particularly when the disease presents with atypical features such as isolated necrotic mediastinal lymphadenopathy.

Importantly, melioidosis can closely mimic tuberculosis both clinically and radiologically, contributing to frequent misdiagnosis in endemic regions. *B. pseudomallei* can induce granulomatous inflammation and, in some instances, caseous necrosis, reproducing the hallmark histopathological features of tuberculous lymphadenitis [4, 5]. Clinically, both diseases may present with prolonged fever, weight loss, cough, mediastinal lymphadenopathy, and pulmonary nodularity. Radiologically, features such as necrotic lymph nodes, tree-in-bud nodules, and patchy infiltrates can be indistinguishable between the two conditions [6, 7]. Additionally, diabetes one of the strongest risk factors for melioidosis is also a known risk factor for tuberculosis, further increasing diagnostic complexity in regions where both pathogens are endemic.

Another important consideration is that tuberculosis and melioidosis rarely can co-infect the same patient, particularly in individuals with impaired immunity such as those with diabetes [8–10]. Co-infection may also influence the clinical course, complicate radiological interpretation, and impact therapeutic decisions. Therefore, establishing a clear microbiological diagnosis becomes crucial in avoiding inappropriate monotherapy, which could worsen outcomes if the second pathogen is not recognised.

In our case, the diagnosis of melioidosis was supported by dual microbiological confirmation from two independent sites sputum culture and EBUS-guided lymph node aspiration [11]. Confirming the organism from both a respiratory sample and the involved lymph nodes provided robust evidence against tuberculosis and ruled out the possibility of a coincidental colonisation or contamination. This multi-site confirmation is particularly important in TB-endemic regions, where reliance on imaging alone can be misleading and where co-infection remains a real possibility. Obtaining microbiological evidence from more than one site therefore strengthens diagnostic certainty and ensures timely initiation of appropriate therapy.

Though lymphadenitis can occasionally be observed in melioidosis, necrotic mediastinal lymphadenopathy as the predominant or sole presenting feature remains exceptionally rare in the literature [12–14]. In tuberculosis-endemic settings, this often leads clinicians to prioritise TB, potentially delaying targeted therapy for melioidosis. This case highlights the need for systematic microbiological evaluation including cultures and tissue sampling rather than assuming tuberculosis based on radiological pattern alone.

Treatment typically involves two to eight weeks of intravenous antibiotics (e.g. meropenem or ceftazidime), followed by an extended eradication phase of at least 3–4 months with oral co-trimoxazole [15]. Adherence to this prolonged regimen is essential to prevent relapse, a known complication when therapy is incomplete.

In conclusion, melioidosis though uncommon should be strongly considered in individuals from endemic regions who present with unexplained fever and necrotic mediastinal lymphadenopathy, particularly in those with diabetes. Its clinical and radiological resemblance to tuberculosis, along with the possibility of co-infection, underscores the need for definitive microbiological confirmation. Early recognition and appropriate therapy remain crucial to improving outcomes in this potentially fatal but treatable infection.
